# Survival outcomes according to the tumor location and prognostic factor in metastatic rectal cancer: a multicenter retrospective cohort study

**DOI:** 10.3389/fonc.2024.1363305

**Published:** 2024-06-14

**Authors:** Olcun Umit Unal, Seval Akay, Huseyin Salih Semiz, Murat Keser, Gonul Demir, Zeliha Guzeloz Capar, Erkut Demirciler, Tugba Yavuzsen, Serkan Degirmencioglu, Bilgin Demir, Esin Oktay, Meltem Demirtas Gulmez, Mehmet Emin Arayici

**Affiliations:** ^1^ Department of Medical Oncology, Tepecik Education and Research Hospital, Izmir, Türkiye; ^2^ Department of Medical Oncology, Izmir Faculty of Medicine, University of Health Sciences, Izmir, Türkiye; ^3^ Department of Internal Medicine, Division of Medical Oncology, Faculty of Medicine, Dokuz Eylul University, Izmir, Türkiye; ^4^ Department of Radiation Oncology, Tepecik Education and Research Hospital, Izmir, Türkiye; ^5^ Department of Medical Oncology, Denipol Private Hospital, Denizli, Türkiye; ^6^ Department of Medical Oncology, Ataturk State Hospital, Aydin, Türkiye; ^7^ Department of Medical Oncology, Adnan Menderes University, Aydin, Türkiye; ^8^ Department of Biostatistics and Medical Informatics, Faculty of Medicine, Dokuz Eylul University, Izmir, Türkiye; ^9^ Department of Public Health, Faculty of Medicine, Dokuz Eylul University, Izmir, Türkiye

**Keywords:** metastatic rectal cancer, primary tumor site, prognostic factor, survival, multiple metastatic site

## Abstract

**Background & aims:**

Prognostic factors of metastatic rectal cancer are not well known. We aim to determine prognostic factors affecting survival for metastatic rectal cancer patients and also to investigate the effect of tumor localization on overall survival.

**Methods:**

Metastatic rectal cancer patients who received treatment in 5 different centers between 2012 and 2022 were included. Prognostic factors for survival were evaluated using univariate and multivariate analysis. The statistical methods included Pearson’s chi-square test, Fisher exact test, Log-rank test, and Cox regression model.

**Results:**

A total of 283 patients with metastatic rectal cancer were included in the study. The median OS was not significantly different among the three groups (upper rectum 30.1 months, middle rectum 28.3 months, and low rectum cancer 24.8 months; log-rank p = 0.25). In univariate analysis, Grade 3, ECOG performance status 2, the presence of multiple metastatic sites, the presence of KRAS mutation, the presence of liver metastases, the presence of nonregional lymph node metastases, and the presence of bone metastases were significant predictors of poor survival. In multivariate analysis, Grade 3, ECOG performance status 2, and the presence of multiple metastatic sites were determined as indicators of worse prognosis.

**Conclusion:**

Our findings, primary tumor location did not affect survival in metastatic rectal cancer. The most important factors affecting survival were multiple metastatic sites, tumor grade, and ECOG performance status.

## Introduction

Colorectal cancer is the 3rd most frequently diagnosed cancer in the world for both sexes, and it is the 3rd most common cause of death from cancer (1). About one-third of all colorectal cancers are rectal cancer (2). The definition of colon and rectal cancer as one or two different entities is still controversial. Both colon and rectal cancer have similar etiological, precancerous lesions and spread (3). However, colon and rectal cancers show differences in terms of gender, age, tumor progression, metastatic site, and adjuvant treatments, for example, lung and bone metastases are detected more frequently in rectal cancer than in colon cancer (2, 4).

Rectal cancer is not a single disease but differs biologically and anatomically. Rectal cancer is anatomically divided into three: upper, middle, and lower (10–15 cm, 5–10 cm, 0–5 cm, respectively, from the anal verge) (5). Surgery is the standard treatment after neoadjuvant chemoradiotherapy in stage 2–3 of rectal cancer. On the other hand, stage 2–3 upper rectal cancers have similar and better survival with left-sided colon cancers, while lower and middle rectal cancers have a worse prognosis and survival (6).

Metastatic rectal cancer is generally treated with a similar treatment modality (chemotherapy and biologic agent) to left colon tumors. However, there is no study investigating the survival of upper, middle, and lower rectal tumors in the metastatic stage. This study aimed to evaluate the survival and prognosis of anatomical differences and other factors in metastatic rectal cancer.

## Methods

In this study, metastatic rectal cancer patients who received treatment in 5 different centers between January 1, 2012, and December 31, 2022 were included and evaluated retrospectively. Data collection started on January 1, 2023, and data was last processed until May 31, 2023. Study inclusion criteria: Being over 18 years old and being diagnosed with metastatic rectal cancer. The patient’s clinical information was obtained from medical records. Distal, middle, and upper-rectal cancers were defined as tumors located less than 5.1, 5.1 to 10, and 10.1 cm or more above the anal verge. The chi-square and Fisher’s exact tests were used in the analysis of categorical variables according to their suitability. The Kaplan-Meier method was utilized to estimate the overall survival (OS). OS was defined as the time from the first day of metastasis to the date of death or last seen. Differences in survival were investigated using the log-rank test. Median follow-up times in the research group were quantified using the reverse Kaplan-Meier. Univariate and multivariate Cox proportional hazard regression modeling was applied to identify the best predictive variables to evaluate the effects of the yielded clinical data on survival in patients with rectal carcinoma. The data were evaluated and visualized using the SPSS (v29.0) package program. Two-tailed p < 0.05 was deemed an indicator of statistical significance in all tests performed.

## Results

283 metastatic rectal cancers were included in the study. At the time of diagnosis, 159 (56%) of the patients were stage 4. Kras, Nras, and Braf mutations, and MSI status were examined in 273, 252, 246, and 164 patients, respectively. Kras, Nras, and Braf mutations were detected in 112 (39.6%), 12 (4.4%), and 3 (1.1%) patients, respectively. Also, MSI high status was detected in 4 patients(%1.3). Metastasectomy was not performed in any patient. Patients demographic, clinical, and pathological characteristics are shown in **Table 1**.

**Table 1:** Patient, tumor and treatment characteristics.

Characteristics	N(%)
Sex	
Male	190(%67.1)
Female	93(%32.9)
ECOG performance score	
0–1	263(%92.9)
2	20(%7.1)
Comorbidity	
Absent	154(%54.4)
Present	129(%45.6)
Primary tumor localization	
Upper	87(%30.8)
Middle	62(%21.9)
Low	134(%47.3)
Histology	
Adenocarcinoma	262(%92.6)
Mucinous adenocarcinoma	17(%6)
Signet-ring cell carcinoma	4(%1.4)
Diferantation	
Low	79(%27.9)
Moderate	90(%31.8)
Poor	18(%6.4)
Unknown	96(%33.9)
Number of metastatic sites	
1	142)%50.2)
2	93(%32.9)
≥3	48(%%16.9)
Hepatic metastasis	
Present	165(%58.3)
Absent	118(%41.7)
Pulmonary metastasis	
Present	167(%59)
Absent	116(%41)
Peritoneal metastasis	
Present	37(%13.1)
Absent	246(%86.9)
Bone metastasis	
Present	35(%12.4)
Absent	248(%87.6)
Ovarian metastasis	
Present	2(%0.7)
Absent	281(%99.3)
Nonregional lymph node metastasis	
Present	66(%23.3)
Absent	217(%76.7)
Brain metastasis	
Present	8(%2.8)
Absent	275(%97.2)
First line regimen	
Chemotherapy^x^+ bevasizumab	127(%45)
Chemotherapy^x^-antiEGFR	86(%30.1)
Chemotherapy	65(%23.1)
Missing	5(%1.8)
Second line	
Chemotherapy^x^-bevasizumab	61(%21.6)
Chemotherapy^x^	45(%16)
Chemotherapy^x^-antiEGFR	35(%12.4)
FOLFIRI-aflibercept	23(%8)
Missing	119(%42)

X=FOLFOX or FOLFIRI.

While the majority of patients received first-line treatment, the rate of those receiving second-line treatment was 58%. The majority of patients had received bevacizumab-based chemotherapy in first-line therapy. In addition to, VEGF-based therapies were used more frequently than EGFR-based therapies in second-line therapy. Details of the treatment regimens are presented in detail in **Table 1**.

**Figure 1:**
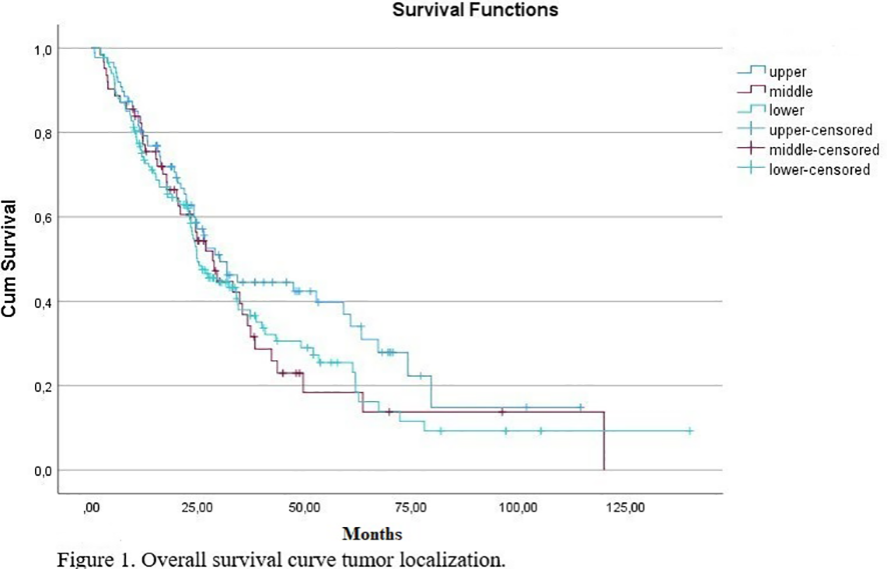
Overall survival curve tumor localization.

The median OS was not significantly different among the three groups (upper rectum 30.1 months, middle rectum 28.3 months, and low rectum cancer 24.8 months; log-rank p = 0.25; **Figure 1**). In univariate analysis, Grade 3 (according to 1–2) (p = 0.002), ECOG performance status 2 (according to 0–1) (p < 0.001), presence of multiple metastases (p < 0.001), presence of KRAS mutation (p = 0.022), presence of liver metastases (p = 0.012), and presence of nonregional lymph node metastases (p = 0.001), and presence of bone metastases (p = 0.001) were associated with worse OS. Univariate analysis results are summarized in **Table 2**. In multivariate analyses, Grade 3 (based on 1–2) (p < 0.001), ECOG performance status 2 (according to 0–1) (p = 0.030), and the presence of multiple metastatic sites (>2) (p < 0.001) were associated with worse OS (**Table 3**).

**Table 2:** Univariate analysis of clinical parameters in patients with rectal carcinoma.

Parameter	Overall Survival	
	HR (95% CI)	*p value*
Age (years)	0.999 (0.986–1.013)	0.902
Sex		
Female	1	0.859
Male	0.972 (0.711–1.330)	
Stage at the time of diagnosis		
1–2		0.054
3–4	11.711 (0.990–2.957)	
GRADE		
1–2	1	**0.002**
3	2.443 (1.383–4.315)	
Tumor localization		
Upper	1	0.098
Middle**/**Lower	1.316 (0.950–1.823)	
ECOG performance status		
0–1	1	**< 0.001**
2	2.397 (1.446–3.976)	
Number of metastatic sites		
1–2	1	**< 0.001**
> 2 (3,4,5,6)	1.947 (1.360–2.788)	
KRAS mutation		
No	1	**0.022**
Yes	1.412 (1.051–1.897)	
NRAS mutation		
No	1	0.560
Yes	1.236 (0.607–2.514)	
Liver metastasis		
No	1	**0.012**
Yes	1.476 (1.089–2.001)	
Lung metastasis		
No	1	0.317
Yes	1.167 (0.862–1.579)	
Bone metastasis		
No	1	**0.001**
Yes	1.948 (1.297–2.925)	
Nonregional lymph node metastasis		
No	1	**0.001**
Yes	1.743 (1.245–2.441)	
Brain metastasis		
No	1	0.381
Yes	1.491 (0.610–3.648)	

HR, hazard ratio; CI, confidence interval; ECOG, Eastern Cooperative Oncology Group.

Significant p values are indicated in bold.

**Table 3:** Multivariate analyses of clinical parameters in patients with rectal carcinoma.

Parameter	OOver Overall Overall Survival	
	HR (95% CI)	*p value*
Age (years)	0.991 (0.974–1.009)	0.324
Sex		
Female	1	0.100
Male	0.700 (0.458–1.071)	
Stage at the time of diagnosis		
1–2	1	0.502
3–4	1.239 (0.663–2.316)	
GRADE		
1–2	1	**< 0.001**
3	2.768 (1.524–5.028)	
Tumor localization		
Upper	1	0.244
Middle**/**Lower	1.286 (0.842–1.963)	
ECOG performance status		
0–1	1	**0.030**
2	2.114 (1.074–4.161)	
Number of metastatic sites		
1–2	1	**< 0.001**
> 2 (3,4,5,6)	2.582 (1.637–4.073)	
Liver metastasis		
No	1	0.700
Yes	1.084 (0.718–1.637)	
KRAS mutation		
No	1	0.143
Yes	1.336 (0.907–1.967)	

HR, hazard ratio; CI, confidence interval; ECOG, Eastern Cooperative Oncology Group.

Significant p values are indicated in bold.

## Discussion

In this study, which examined 283 patients with metastatic rectal cancer who were followed up and treated in 5 oncology clinics, survival was investigated in terms of tumor location and it was found that tumor location (upper, middle, lower) did not show any difference in overall survival. It has been shown in more than one study in the literature that upper rectum tumors have better survival and prognosis compared to lower and middle rectal tumors in stage 1–3 rectal cancers (5–7). On the other hand, in a study comparing Kras mutant metastatic colorectal cancer patients according to tumor location, it was found that the worst survival was in the rectum region compared to right and left colon cancers (8). In our study, survival according to localization varies between 24 and 30 months and is comparable to survival in previous studies (7–9).

In our study, we present data on the distribution of rectal cancer metastases in a real-life group of patients, the majority of whom were treated with novel oncological therapies, and describe their prognostic value on clinical outcomes. In particular, assessing the impact of tumor burden and metastatic sites on prognosis is difficult and has been performed in a few studies. Shida et al. found that detecting metastasis in more than one area was a poor prognostic factor (10). Similarly, in the study of Ge et al., it was found that the prognosis of multiple metastases was poor (11). In our study, more than two metastases were found to be poor prognostic, confirming previous studies. In more than one study, lung metastasis is a good prognosis indicator in rectal cancer, and in our study, it was found to not affect the prognosis (12, 13). In many studies, liver, bone, and non-regional lymph node metastases are worse prognostic than other metastases (9, 11, 12). In our study, we found that these metastatic regions were poor prognostic.

In our study, in multivariate analysis, two of the three most important prognostic factors affecting survival were determined to be ECOG performance status and tumor grade. These two prognostic factors have been identified in numerous studies as important prognostic factors for both rectal cancer and other cancers (14–16).

Angiogenesis is a universal requirement for colorectal cancers to grow beyond the limitations of oxygen diffusion through the existing vasculature (17, 18). Inhibition of angiogenesis has proven beneficial in the treatment of many types of malignancies, including colorectal cancer (19, 20). Bevacizumab and aflibercept were used for VEGF inhibition in most of the patients included in this study, which may have affected our survival.

It has been shown in many studies that primary tumor surgery and metastasectomy prolong survival in metastatic rectal cancer (21–24). This issue was not evaluated in our study and was one of its most important limitations.

When interpreting the results of our study, it should be taken into account that it has numerous limitations. Since the entire group was not treated with the same chemotherapy regimens, response rates, and survival may have been affected. There was a lack of information regarding NRAS, BRAF mutation, and MSI status, and due to this missing information, we cannot evaluate the impact of the relevant patient group on our data. The main reason for this is that these biomarkers have only recently become clinically relevant for treatment decisions and can therefore be studied from now on. Additionally, due to the small number of patients, we could not evaluate the effectiveness of antiVEGF and antiEGFR agents according to the difference in rectum localization.

## Conclusion

As a result, in this study, it was determined that primary tumor location did not affect survival in metastatic rectal cancer, and the most important factors affecting survival were tumor burden, tumor grade, and ECOG performance score. It was found that liver, bone, and non-regional lymph node metastases had a worse prognosis, and lung and brain metastases did not affect the prognosis.

## Data availability statement

The raw data supporting the conclusions of this article will be made available by the authors, without undue reservation.

## Ethics statement

The studies involving humans were approved by tepecik education and research hospital. The studies were conducted in accordance with the local legislation and institutional requirements. Written informed consent for participation was not required from the participants or the participants’ legal guardians/next of kin because It was not included because it was a retrospective file scan.

## Author contributions

OU: Writing – original draft, Writing – review & editing. SA: Writing – original draft, Writing – review & editing. HS: Data curation, Writing – original draft, Writing – review & editing. MK: Data curation, Writing – original draft, Writing – review & editing. GD: Data curation, Writing – original draft, Writing – review & editing. ZG: Data curation, Writing – original draft, Writing – review & editing. ED: Data curation, Writing – original draft, Writing – review & editing. TY: Data curation, Writing – original draft, Writing – review & editing. SD: Data curation, Writing – original draft, Writing – review & editing. BD: Data curation, Writing – original draft, Writing – review & editing. EO: Data curation, Writing – original draft, Writing – review & editing. MD: Data curation, Writing – original draft, Writing – review & editing. MA: Formal analysis, Writing – original draft, Writing – review & editing.

## References

[1] Siegel RL Wagle NS Cercek A Smith RA Jemal A. Colorectal cancer statistics, 2023. CA Cancer J Clin. (2023) 73:233–54. doi: 10.3322/caac.21772 36856579

[2] Wang CB Shahjehan F Merchea A Li Z Bekaii-Saab TS Grothey A. Impact of tumor location and variables associated with overall survival in patients with colorectal cancer: A mayo clinic colon and rectal cancer registry study. Front Oncol. (2019) 9:76. doi: 10.3389/fonc.2019.00076 30838175 PMC6389639

[3] Koca D Ünal OÜ Öztop I Yılmaz U. FOLFOX7 regimen in the first-line treatment of metastatic colorectal cancer. Turk J Gastroenterol. (2014) 25:198–204. doi: 10.5152/tjg.2014.3609 25003682

[4] Uncu D Aksoy S Çetin B Yetişyiğit T Özdemir N Berk V. Results of adjuvant FOLFOX regimens in stage III colorectal cancer patients: retrospective analysis of 667 patients. Oncology. (2013) 84:240–5. doi: 10.1159/000336902 23392240

[5] Chiang JM Hsieh PS Chen JS Tang R You JF Yeh CY. Rectal cancer level significantly affects rates and patterns of distant metastases among rectal cancer patients post curative-intent surgery without neoadjuvant therapy. World J Surg Oncol. (2014) 30:12:197. doi: 10.1186/1477-7819-12-197 PMC4108971 24980147

[6] Cheng L Chen J Chen S Wei S Han S. Distinct prognosis of high versus mid/low rectal cancer: a propensity score-matched cohort study. J Gastrointest Surg. (2019) 23:1474–84. doi: 10.1007/s11605-018-04072-1 30617772

[7] Rosenberg R Maak M Schuster T Becker K Friess H Gertler R. Does a rectal cancer of the upper third behave more like a colon or a rectal cancer? Dis Colon Rectum. (2010) 53:761–70. doi: 10.1007/DCR.0b013e3181cdb25a 20389210

[8] Lee K Chen W Jiang J Yang S Wang H Chang S. The efficacy of anti-EGFR therapy in treating metastatic colorectal cancer differs between the middle/low rectum and the left-sided colon. Br J Cancer. (2021) 125:816–25. doi: 10.1038/s41416-021-01470-2 PMC8437976 34188197

[9] Chen T Chen W Jiang J Yang S Wang H Chang S. Effect of primary tumor location on postmetastasectomy survival in patients with colorectal cancer liver metastasis. J Gastrointest Surg. (2021) 25:650–61. doi: 10.1007/s11605-020-04855-5 33201458

[10] Shida D Inoue M Tanabe T Moritani K Tsukamoto S Yamauchi S. Prognostic impact of primary tumor location in Stage III colorectal cancer-right-sided colon versus left-sided colon versus rectum: a nationwide multicenter retrospective study. J Gastroenterol. (2020) 55:958–68. doi: 10.1007/s00535-020-01706-7 32651860

[11] Ge H Zhou Z Li Y Wang D Gungor C. Prognostic factors and individualized nomograms predicting overall survival in stage IV rectal cancer patients with different metastatic status: a SEER-based study. Transl Cancer Res. (2022) 11:3141–55. doi: 10.21037/tcr PMC9552055 36237239

[12] Muzellec L Campion L Bachet JB Taieb J Fremont E Senellart H. Prognostic score for synchronous metastatic rectal cancer: a real-world study. Dig Liver Dis. (2023) 55:1411–6. doi: 10.1016/j.dld.2023.03.004 37005173

[13] Wells SM Boothe D Ager BJ Tao R Gilcrease GW Lloyd S. Analysis of nonsurgical treatment options for metastatic rectal cancer. Clin Colorect Cancer. (2020) 19:91–9. doi: 10.1016/j.clcc.2019.11.002 32173281

[14] Akdeniz N Küçüköner M Kaplan MA Urakçı Z Sezgin Y Karhan O. Survival impact of optimal treatment for elderly patients with colorectal cancer:A real world study. Indian J Cancer. (2021) 58:539–44. doi: 10.4103/ijc.IJC_409_19 34380826

[15] Ozyukseler DT Basak MT Ay S Koseoglu A Arıcı S Oyman A. Prognostic factors of ado-trastuzumab emtansine treatment in patients with metastatic HER-2 positive breast cancer. J Oncol Pharm Pract. (2021) 27:547–54. doi: 10.1177/1078155220924088 32423326

[16] Fan D Li X Yu Y Wang X Fang J Huang C. Correlation between clinicopathological characteristics and the clinical prognosis of patients with gastroenteropancreatic neuroendocrine tumors. Mol Clin Oncol. (2023) 19:85. doi: 10.3892/mco 37809346 PMC10557094

[17] Bisgin A Kargi A Yalcin AD Aydin C Ekinci D Savas B. Increased serum sTRAIL levels were correlated with survival in bevacizumab-treated metastatic colon cancer. BMC Cancer. (2012) 12:58. doi: 10.1186/1471-2407-12-58 22313795 PMC3359245

[18] Celik B Yalcin AD Bisgin A Dimitrakopoulou-Strauss A Kargi A Strauss LG. Level of TNF-related apoptosis-inducing-ligand and CXCL8 correlated with 2-[18F]Fluoro-2-deoxy-D-glucose uptake in anti-VEGF treated colon cancers. Med Sci Monit. (2013) 19:875–82. doi: 10.12659/MSM.889605 PMC3808407 24145180

[19] Yalcin AD Kargi A Gumuslu S. Blood eosinophil and platelet levels, proteomics patterns of trail and CXCL8 correlated with survival in bevacizumab treated metastatic colon cancers. Clin Lab. (2014) 60:339–40. doi: 10.7754/Clin.Lab.2013.130425 24660552

[20] Jia SN Han YB Yang R Yang ZC. Chemokines in colon cancer progression. Semin Cancer Biol. (2022) 86:400–7. doi: 10.1016/j.semcancer.2022.02.007 35183412

[21] Ho MF Lai VC Ng DCK Ng SSM. Prognosis of patients with unresectable stage IV Colon cancer undergoing primary tumor resection: A multicenter study of minimally symptomatic or asymptomatic primary tumor. Asian J Surg. (2023) 46:3710–5. doi: 10.1016/j.asjsur.2022.11.127 36522225

[22] Dai S Zhao W Yue L Qian X. A competing risk for nomogram of the role of metastasectomy in patients with colorectal cancer and liver metastases. Asian J Surg. (2023) 46:2468–71. doi: 10.1016/j.asjsur.2022.12.066 36567218

[23] Park J Baik H Kang SH Seo SH Kim KH Oh MK. Comparison between oxaliplatin therapy and capecitabine monotherapy for high-risk stage II - III elderly patients with colon cancer. Asian J Surg. (2022) 45:448–55. doi: 10.1016/j.asjsur.2021.07.067 34364765

[24] Ishiyama Y Tachimori Y Harada T Mochizuki I Tomizawa Y Ito S. Oncologic outcomes after laparoscopic versus open multivisceral resection for local advanced colorectal cancer: a meta-analysis. Asian J Surg. (2023) 46:6–12. doi: 10.1016/j.asjsur.2022.02.047 35568616

